# Prevalence of Dyslipidemia in Ischemic Stroke Patients: A Single-Center Prospective Study From Pakistan

**DOI:** 10.7759/cureus.25880

**Published:** 2022-06-12

**Authors:** Raja Sheraz Ullah Khan, Mehwish Nawaz, Sarfaraz Khan, Hassan Ali Raza, Talha Nazir, Muhammad Saad Anwar, Hafiz Muhammad Faisal Nadeem, Zia Ur Rehman, Amina Akram

**Affiliations:** 1 Internal Medicine, Shalamar Hospital, Lahore, PAK; 2 Internal Medicine, Hayatabad Medical Complex, Peshawar, PAK; 3 Internal Medicine, Primary Health Care Corporation, Gujranwala, PAK; 4 Neurology, AINeuroCare Academy, Dallas, USA; 5 Neurology, AINeuroCare, Dallas, USA; 6 Pulmonary and Critical Care, District Headquarter Hospital, Lahore, PAK; 7 Cardiology, Punjab Institute of Cardiology, Lahore, PAK

**Keywords:** hypertension, diabetes mellitus, risk factor, dyslipidemia, : ischemic stroke

## Abstract

Background

*Stroke* is a debilitating condition that adds morbidity to the patient and is an economic burden to society. Several modifiable risk factors can be identified and controlled, and dyslipidemia is one such under-diagnosed and least reported risk factor in Pakistan. We aimed to conduct this study to determine the frequency of dyslipidemia in ischemic stroke patients.

Methodology

We conducted a prospective cross-sectional study for seven months at Shalamar Hospital, Lahore, from November 2020 to May 2021. One hundred four patients were enrolled as per inclusion and exclusion criteria. After informed consent, a blood sample was taken from the patients and sent to a laboratory to determine dyslipidemia. Demographic details, history, and co-morbidities were also noted on a performa. All the collected data were analyzed in SPSS 20.0.

Results

The mean age was 53.09 ± 12.51 years. Of 104 patients, 60 (57.69%) were male, and 44 (42.31%) were females, with a male-to-female ratio of 1.4:1. The mean duration of disease in our study was 5.77 ± 3.33 hours. The mean BMI was 27.54 ± 3.03 kg/m2. In our study, the frequency of dyslipidemia in ischemic stroke patients was 39.42% (41 patients).

Conclusion

This study concluded that dyslipidemia in ischemic stroke patients is very high in the Pakistani population. This highlighted the need to control this modifiable risk factor in the population. Strategic measures, including pharmacological therapy and diet modification, should be adopted, and awareness about the disease burden, control, and importance should be spread.

## Introduction

Stroke is the third leading cause of mortality globally, and 25.7 million people around the globe have survived a stroke. Stroke may be because of ischemic etiology or may result from hemorrhagic infarction. The most common etiology of stroke is the ischemic world widely [1]. Stroke is a debilitating condition contributing to morbidity and mortality as the most common neurological cause [2]. A recent stroke survey highlighted the disease's burden and morbidity and reported that almost 33% of all affected individuals develop permanent disability [3]. The prevalence of stroke is high in Asia (including Pakistan) due to the high burden of vascular risk factors, with an estimated 250 persons suffering from a stroke in one lac Pakistani population [4]. Stroke is mostly the disease of older people and comes up with dementia, further contributing to its morbidity. This demands stroke control in the elderly to help improve their quality of life [4].

There is a role of various factors that are responsible for a stroke. Some of these are modifiable, whereas others are non-modifiable. We can control modifiable risk factors only. A 2014 study found a significant incidence of modifiable risk factors in the Pakistani population, including hypertension and dyslipidemia. According to the same study, atherosclerosis is the most prevalent cause of this problem. Elderly individuals had a greater risk-adjusted mortality rate and a higher rate of complications and hospitalization length [5].

It is critical to identify and adjust the risk factors to reduce the occurrence of stroke. Diabetes mellitus, hypertension, cigarette smoking, dyslipidemia, and valvular heart disease are major modifiable risk factors [5]. Understanding the comparison between each risk factor can assist clinicians in making sound illness management decisions. Previous research has shown that dyslipidemia is more likely in people with ischemic stroke [6,7].

Research indicated that dyslipidemia in individuals with ischemic stroke was around 15.6% [8], whereas a local investigation found it at 37.1% [9]. Subburaj et al. have shown a much more significant percentage (65.7%) of dyslipidemia in ischemic stroke patients [10]. In Pakistan, dyslipidemia is a very under-reported cause of stroke due to a lack of central registry and limited resources in the third world, requiring laboratory investigations to diagnose dyslipidemia. A recent study noticed 30% dyslipidemia in stroke patients, which contrasts with what was reported by Pinzon et al. (81%) [11,12].

The frequency of stroke is on the rise in Pakistan. Dyslipidemia being identified as a common modifiable risk factor for ischemic stroke implies that identifying dyslipidemia in ischemic stroke patients is essential. Because earlier research yielded inconsistent results across different demographic locations, we aimed to determine the prevalence of dyslipidemia in Ischemic Stroke in the local community. This problem identification will lay the foundation for policies and actions necessary by the national control program to minimize the disease burden.

## Materials and methods

This prospective descriptive cohort study was conducted at Shalamar Hospital, Lahore, after getting permission from the institutional review board Shalamar Medical and Dental College, Lahore (IRB) with IRB number CPSP/REU/MED-2016-073-11097. The study duration was seven months, from November 2020 to May 2021. A sample size of 104 was calculated with an anticipated proportion of dyslipidemia of 15.6% in ischemic stroke patients [8]. Patients of both genders, ages 25 to 75 years, who had recently suffered from ischemic stroke were included in the study.

All patients suffering from hemorrhagic stroke as determined by Computerized tomography (CT) Scan reporting, on lipid-lowering drugs, alcoholics, or having any chronic kidney and liver disease were excluded from the study.

A total of 104 patients presented with ischemic stroke following the inclusion and exclusion criteria were selected. An informed consent stating details and benefits of the study was signed by either the participant or a close relative. The patient's history (lifestyle, smoking, duration of disease), demographic features like age, gender, etc., and co-morbidities like diabetes and hypertension were asked in detail and were recorded on a proforma. A blood sample was taken from the patient and sent to the institution's laboratory to determine the presence or absence of dyslipidemia through a lipid profile. Dyslipidemia was defined as having any one of these i.e, triglyceride (TG) level >150 mg/dl, total cholesterol (TC) >200 mg/dl, low-density lipoprotein (LDL) >130 mg/dl and high-density lipoprotein (HDL) <40 mg/dl.

The data were analyzed using IBM SPSS Statistics for Windows, Version 20.0. (IBM Corp., Armonk, NY). Mean and standard deviation was calculated for age, duration of a stroke, and BMI. Frequencies and percentages were calculated for categorical variables like a place of living, gender, hypertension, diabetes mellitus, lifestyle, smoking, and dyslipidemia. Stratification of the variables was done, and post-stratification Chi-square was applied to see their effect on the frequency of dyslipidemia. P-value ≤ 0.05 was considered significant.

## Results

Of 104 included patients, 60 (57.69%) were male, and 44 (42.31%) were female. The mean age of patients was 53.09 ± 12.51 years, ranging from 25 to 75 years. The majority of the patients, 57 (54.81%), were between 51 to 75 years of age. The mean duration of the disease was 5.77 ± 3.33 hours. The mean body mass index (BMI) was 27.54 ± 3.03 kg/m2. The details of baseline characteristics are given in Table 1.

**Table 1:** Baseline patient characteristics All the baseline characteristics of the patients were included in our study.

Characteristic	Number of Patients (n)	Percentage (%)
Age (in years) 25-50	47	45.19
51-75	57	54.82
Gender Male	60	57.69
Female	44	42.31
Duration of disease (hours) ≤6	72	69.23
>6	32	30.77
BMI <25	53	50.96
≥25	51	49.04
Place of living Rural	39	37.50
Urban	65	62.50
Lifestyle Simple	45	43.27
Sedentary	59	56.73
Diabetes Mellitus Yes	50	48.08
No	54	51.92
Hypertension Yes	67	64.42
No	37	35.58

Dyslipidemia was present in 41 (39.42%) patients. Stratification of dyslipidemia with smoking only showed a statistically significant result (p-value <0.05). All other stratified variables did not have a significant association with dyslipidemia (p-value >0.05). The detailed results of the stratified variables are given along with their p-values in table 2.

**Table 2:** Stratified Analysis Association of the stratified variable with dyslipidemia

Characteristic	Dyslipidemia Present	Dyslipidemia Absent	p-value
Age (in years) 25-50	16	31	0.308
51-75	25	32	
Gender Male	22	38	0.502
Female	19	25	
Duration (in hours) ≤6	26	46	0.300
>6	15	17	
BMI (kg/m^2^) ≤27	19	34	0.447
>27	22	29	
Place of living Rural	17	22	0.501
Urban	24	41	
Lifestyle Simple	21	24	0.187
Sedentary	20	39	
Diabetes Mellitus Yes	21	29	0.605
No	20	34	
Hypertension Yes	25	42	0.554
No	16	21	
Smoking Yes	24	22	0.018
No	17	41	

## Discussion

In this study, we have recorded the prevalence of dyslipidemia in patients with ischemic stroke. As per 2008 Who statistics, the prevalence of dyslipidemia in Southeast Asia was 30.3% [13]. Our study also showed that around 39% of ischemic stroke patients were later diagnosed with dyslipidemia. The results were comparable to another study done in Pakistan, which showed that 55% of the study population presented with ischemic stroke also had dyslipidemia [14].

Another study showed that the outcome of ischemic stroke is associated with abnormal lipid levels with OR of TC >6.22mmol/L (3.013), TG >2.26mmol/L (0.883), LDL-C >4.14mmol/L (3.157) and HDL-C <1.04mmol/L (0.482). Furthermore, calibrated model using the Hosmer Lemeshow goodness of fit test showed no significant difference between observed and predicted outcomes [15]. As dyslipidemia is an important modifiable risk factor for ischemic stroke, early identification can help reduce the prevalence of Ischemic Stroke.

In addition to dyslipidemia, diabetes and hypertension are other known risk factors associated with ischemic stroke [16]. Our study also showed that 48.08% were diabetic, and 64.42% were hypertensive. In our study, this constitutes a higher percentage of these two diseases contributing to stroke. Although dyslipidemia was not associated with Diabetes Mellitus and Hypertension ( p-value > 0.05), smoking history was significantly associated with dyslipidemia increasing the incidence of stroke. A meta-analysis showed a strong association between current smokers (odds ratio 1.92) and former smokers (odds ratio 1.3) with the incidence of stroke and dyslipidemia [17]. The stratified analysis based on smoking was also significant in our study (p-value = 0.018). 

A meta-analysis showed that reducing 1mmol/L LDL cholesterol reduces the risk of stroke by 21.1% [18]. Another meta-analysis showed similar results in reducing the incidence of new events [19] and re-occurrence of stroke [20] using statin therapy. However, it showed no significant reduction in fatal stroke with statin therapy [19].

The patients included in our study were neither taking any lipid-lowering drug nor were they diagnosed with dyslipidemia before the stroke. It is important to take dyslipidemia as a risk factor along with other modifiable risk factors. Multiple meta-analyses have established that reducing LDL cholesterol reduces the overall risk of stroke [20]. Statins are the first-line medications for dyslipidemia, and other options include fibrates, ezetimibe, and PCSK9 inhibitors [21]. 

The limitation of this study is that it was a uni-centered study localized to a specific population. Also, we need studies with a bigger sample size to evaluate the incidence of dyslipidemia. Although the sample size is small, it is probably the most extensive study in Pakistan. The available data on the Pakistani population is inconsistent. So, this study will add value to the existing literature.

## Conclusions

The prevalence of dyslipidemia is high in the Pakistani population. Dyslipidemia is a modifiable risk factor for stroke that should be treated in patients who are at high risk. Early diagnosis and control in the general population will reduce this morbid condition burden, especially among those at higher risk. Lifestyle modification and prescription of lipid-lowering drugs as primary stroke prevention may reduce the incidence of Stroke in Pakistan. Furthermore, more extensive studies are required to assess the prevalence of dyslipidemia so that resources can be allocated appropriately.
